# Supplementary figures and images for: Lectin-Based Affinity Enrichment and Characterization of N-Glycoproteins from Human Tear Film by Mass Spectrometry
Source: Molecules. 2023 Jan 8;28(2):648. doi: 10.3390/molecules28020648 (PMC9864693; doi:10.3390/molecules28020648)

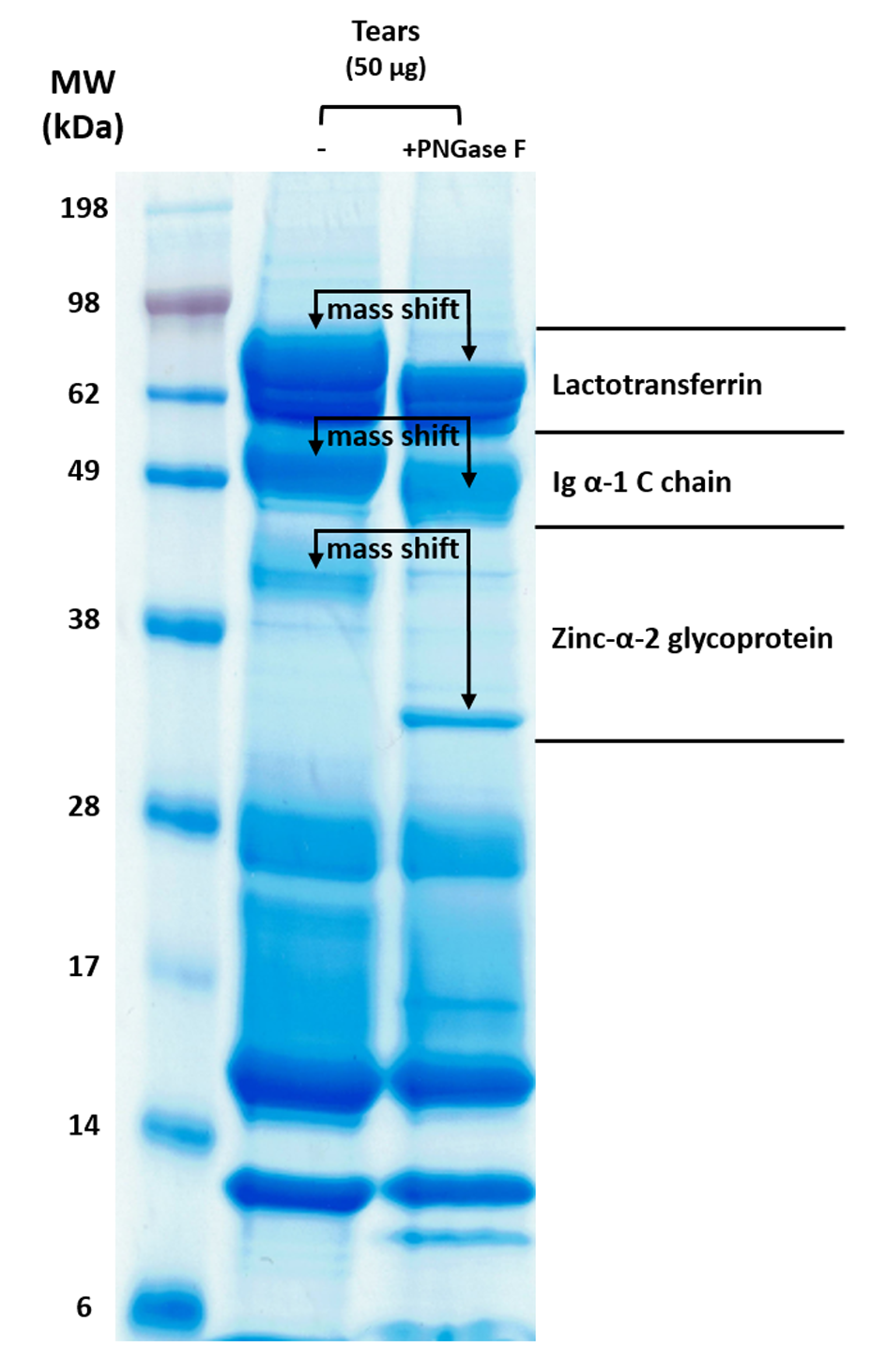

Supplement: Supplementary file 1 [file molecules-28-00648-s001.zip › Figure S1.tif]

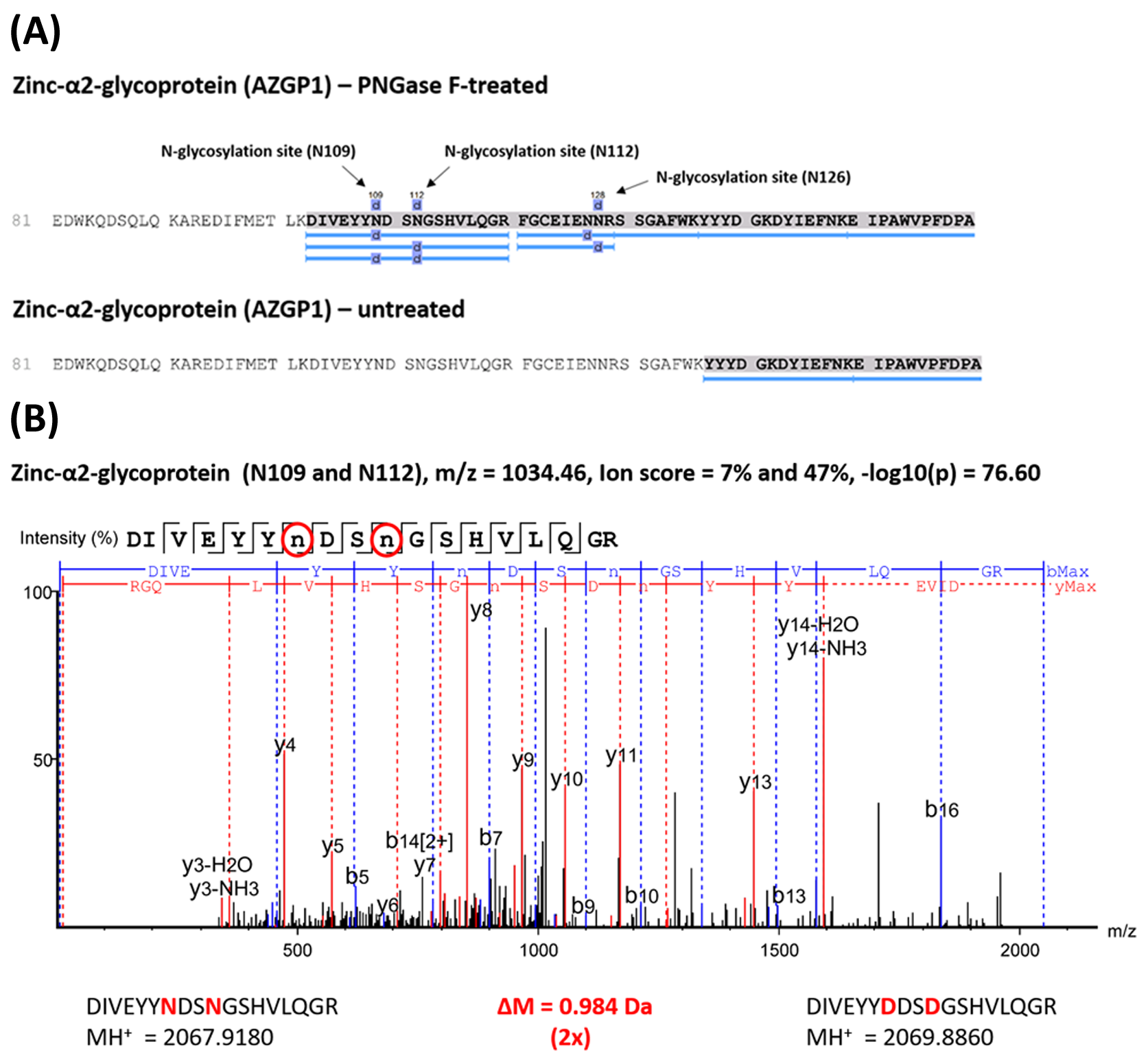

Supplement: Supplementary file 1 [file molecules-28-00648-s001.zip › Figure S2.tif]
